# Fellow-Workers and Their Doings

**Published:** 1914-09-12

**Authors:** 


### ROUND THE WORLD.

### Fellow-Workers and their Doings.

[The Editor Invites Early Information and Contributions Marked "Fellow-Workers."]

AMONGST the large number of eminent medical men who have been summoned to the Colours on the mobilisation of the Territorial medical department, Sir Bertrand Dawson serves as captain in the 2nd City of London Hospital Corps, and is well known as a prominent London Hospital physician. Sir Bertrand Dawson, who was promoted to the responsible post of Physician-in-Ordinary to His Majesty on the death of Sir Francis Laking, is extremely popular in his profession, and although society has many claims on him, always insists on giving up the greater portion of his time to the keen pursuit of professional duties, particularly in the wards of hospitals.

SIR FREDERICK S. EVE, who is also taking his place in the 2nd City of London unit as major, is likewise a London Hospital man and one of our most prominent consulting surgeons. Receiving the honour of knighthood three years ago, Sir Frederick Eve has a very distinguished career to look back upon, in the course of which he has held many important appointments. His name has been freely mentioned as a possible successor to the present occupant of the presidential chair at the Royal College of Surgeons.

STATE medicine is represented by Dr. Arthur Newsholme, who is, of course, the chief medical officer to the Local Government Board, and was formerly Medical Officer of Health for Brighton and Examiner at London University. Dr. Newsholme, who was made a C.B. for his public services in 1912, took the M.D.Lond. in 1881, qualifying for the gold medal. He has been for many years a writer on public health subjects, and his work is generally admitted to have been largely instrumental in the development of our present excellent system of preventive medicine.

SIR J. KINGSTON FOWLER, the senior physician to the "Middlesex" and consulting physician to the Hospital for Consumption, Brompton, etc., has also had to take up his position as lieutenant-colonel in the 3rd London Hospital Corps. He has held many important appointments as consulting physician, and is a lecturer on the Practice of Medicine and Pathology at the "Middlesex." Sir J. Kingston Fowler, who is an Examiner in Medicine for the University of Cambridge, was created a K.C.V.O. in 1910. Besides obtaining this distinction, he became M.A., M.D. Cantab, 1884; D.Sc., 1908; F.R.C.P.Lond., 1886; and M.R.C.S.Eng., 1874. He has done much valuable work in connection with consumption and other lung diseases.

ANOTHER prominent member of the Middlesex Hospital staff who is to serve as lieutenant-colonel of the 3rd London Hospital unit is Sir Alfred Pearce Gould. He is senior surgeon there, and a well-known authority on the operative treatment of cancer. Sir Alfred Pearce Gould became a M.S.Lond. in 1866 and F.R.C.S. in 1877. He was created a K.C.V.O. three years ago.

SIR ALFRED KEOGH, K.C.B., M.D., M.R.C.P., who is supervising the Red Cross work in Belgium, had charge of a general hospital in South Africa during the Boer War. His services at that time were recognised by mention in despatches, and he was awarded the Queen's Medal with four clasps, and in addition the C.B. Subsequently he was for five years Director-General of the Army Medical Service, from which important post he retired in 1909. Sir Alfred Keogh received his medical training at Guy's Hospital and Queen's College, Galway. In addition to his qualifications by examination, he has been made at various times an honorary F.R.C.S. Ireland, honorary F.R.C.S. England, and honorary LL.D. Aberdeen.

THE President of the Royal College of Surgeons, Sir William Watson Cheyne, M.B., F.R.C.S., F.R.S., is lieutenant-colonel of the 4th London Hospital Corps. It will be remembered that he served in South Africa as consulting civil surgeon, and for his work there was mentioned in despatches, receiving a medal, and was made a C.B. A prominent member of the clinical and teaching staff at King's College Hospital, Sir William Watson Cheyne has been a voluminous writer on surgical subjects, in addition to which he has enjoyed for some years a very large consulting practice.

SIR JOHN ROSE BRADFORD, one of the physicians to University College Hospital and post-graduate lecturer at the London School of Clinical Medicine at Greenwich, is attached to the 3rd London Hospital Corps, in which he holds the rank of major. Known to a large circle as a keen investigator in various fields of scientific medicine, and particularly in regard to pharmacology, Sir John Rose Bradford, who won the gold medal at the M.D. London examination in 1889, and subsequently was made a Fellow of the Royal Society, is senior medical adviser to the Colonial Office, and was created a K.C.M.G. in 1911.

SIR VICTOR HORSLEY, F.R.S., F.R.C.S., M.D., holds the rank of captain in the 3rd London Territorial Corps. A member of the staff of the National Hospital for Paralysis and Epilepsy in Queen Square, and University College Hospital, he has been especially responsible for the development of brain surgery in this country. To the general public he has of late years become prominent in connection with the temperance crusade and the women's suffrage movement.

THE famous Leeds School of Surgery is represented by Mr. Mayo Robson, who is lieutenant-colonel in the 3rd London General Hospital. Mr. A. W. Mayo Robson was for many years on the staff of the General Infirmary at Leeds, having been consulting surgeon there since he gave up active hospital work in 1902. For some years he has been well known in London, as at Leeds, and three years ago was made a C.V.O. Among other appointments he holds that of honorary consulting surgeon to the King Edward the Seventh Memorial Hospital, Windsor.

NEUROLOGICAL research also has its representative in the Territorial establishments in the person of Dr. F. Mott, who mobilises as lieutenant-colonel in the 4th London General Hospital. The work of Dr. Mott, who is a member of the Charing Cross Hospital staff, in the field of neurological and medico-psychological investigation is too well known to need emphasis, for he and his assistants at the London County Council laboratories at Claybury have enormously advanced progress therein.

